# Hypoxia tolerance is conserved across genetically distinct sub-populations of an iconic, tropical Australian teleost (*Lates calcarifer*)

**DOI:** 10.1093/conphys/cot029

**Published:** 2013-11-11

**Authors:** Geoffrey M. Collins, Timothy D. Clark, Jodie L. Rummer, Alexander G. Carton

**Affiliations:** 1 Centre for Sustainable Tropical Fisheries and Aquaculture & School of Marine and Tropical Biology, James Cook University, Townsville, QLD 4811, Australia; 2 AIMS@JCU Collaborative Research Program, Townsville, QLD 4810, Australia; 3 Australian Institute of Marine Science, PMB 3, Townsville MC, QLD 4810, Australia; 4 ARC Centre of Excellence for Coral Reef Studies, James Cook University, Townsville, QLD 4811, Australia

**Keywords:** Barramundi, climate change, critical oxygen saturation hypoxia, *Lates calcarifer*, tropical

## Abstract

Tropical coastal systems are particularly prone to periods of environmental hypoxia, which can result from organismal respiration as well as thermal stratification, and may be further exacerbated by anthropogenic disturbances. In this study, we used five genetically distinct sub-populations of Australian barramundi (*Lates calcarifer*) to examine the extent of intraspecific variability in hypoxia tolerance. Fish were maintained at two temperatures (26 or 36°C), representing the seasonal thermal range for this species across its tropical distribution in Australia. All fish maintained a constant oxygen consumption rate  as air saturation of the water decreased from 100% down to a critical oxygen saturation ([O_2_]_crit_) of 15.44 ± 3.20 and 21.07 ± 3.92% (means ± SD) at 26 and 36°C, respectively. Mean [O_2_]_crit_, used as a performance measure of hypoxia tolerance, did not differ between sub-populations. No differences were found for resting  between sub-populations at 26°C, but modest differences were detected between two sub-populations at 36°C (3.36 ± 0.62 and 2.83 ± 0.27 mg O_2_ kg^−1^ min^−1^ for Gladstone and Broome sub-populations, respectively). Resting  was lower for sub-populations at 26°C (1.46 ± 0.26 mg O_2_ kg^−1^ min^−1^) than at 36°C (3.10 ± 0.43 mg O_2_ kg^−1^ min^−1^), with a temperature coefficient (*Q*_10_) of 2.12 ± 0.30. We conclude that both hypoxia tolerance and resting  are conserved across the distribution of barramundi in Australia, which reflects the capacity of this species to cope in environments with large fluctuations in both temperature and dissolved oxygen.

## Abstract (precis)

We used five genetically distinct sub-populations of Australian barramundi (Lates calcarifer) to examine the extent of intraspecific variability in hypoxia tolerance at typical (26°C) and warm (36°C) temperatures. Critical oxygen tension ([O2]crit) was lower at 26°C than at 36°C, indicating greater hypoxia tolerance at the cooler temperature, but was not different between sub-populations.

## Introduction

The availability of dissolved oxygen (DO) in aquatic systems is critical in defining the abundance and distribution of biological communities (Pearson *et al.*, 2003). In freshwater and estuarine systems, oxygen can fluctuate between normoxia and severe hypoxia due to respiration of organisms inhabiting such systems and thermal stratification (Pearson *et al.*, 2003; Brauner and Val, 2005). In tropical regions, initial rains at the onset of the monsoon season can carry high loads of organic material into freshwater and estuarine systems, causing a rapid depletion of oxygen, which can be fatal for resident animal populations (Bishop, 1980; Townsend *et al.*, 1992; Erskine *et al.*, 2005). Furthermore, natural occurrences of hypoxia may be exacerbated by anthropogenic disturbances, such as agriculture and urbanization (Pollock *et al.*, 2007).

Altered weather patterns resulting from climate change have the potential to exacerbate periods of environmental hypoxia. Higher water temperatures reduce the solubility of oxygen in water, while concurrently increasing the metabolic demand for oxygen in aquatic ectotherms (Weiss, 1970; Diaz and Breitburg, 2009). As such, the unprecedented rate of temperature rise predicted under current climate change scenarios may push many aquatic species close to, or above, their upper thermal thresholds (Tewksbury *et al.*, 2008; Morrongiello *et al.*, 2011; Huey *et al.*, 2012). This may be particularly pronounced at very low latitudes near the equator, where species may be adapted to mean temperatures that may span only 1–2°C annually (Tewksbury *et al.*, 2008). Thus, it is likely that the elevated temperatures predicted to occur in the future will progressively increase the prevalence and severity of hypoxia in aquatic systems, with flow-on effects to larger-scale processes, such as population growth, fitness and ecosystem dynamics (Pollock *et al.*, 2007).

Fish can respond physiologically and behaviourally to fluctuating DO, although such responses are highly dependent on species and context. For example, fish that encounter hypoxic conditions may actively avoid areas of low DO (Herbert and Steffensen, 2005; Poulsen *et al.*, 2011), increase gill ventilation volume (Jesse *et al.*, 1967; Fernandes and Rantin, 1989), increase the number of red blood cells and haemoglobin concentration (Wells *et al.*, 1989), and/or induce bradycardia (Randall and Smith, 1967). Exposure to acute or chronic hypoxia can have sub-lethal effects, such as altered behaviour and reduced growth and reproduction, which may be further exacerbated by increasing water temperatures (Vaquer-Sunyer and Duarte, 2008).

The physiological responses to hypoxia are regarded as being dependent on the frequency and severity of the hypoxic event (Farrell and Richards, 2009). Below a certain DO, most fish are unable to regulate their oxygen consumption rate 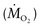 independently of ambient oxygen levels and consequently enter a state of oxygen conformity (Prosser and Brown, 1961; Fernandes *et al.*, 1995; Schurmann and Steffensen, 1997). The level of DO at which this conformity occurs is referred to as the critical oxygen saturation ([O_2_]_crit_) and is commonly used as a measure of the hypoxia tolerance of a species (Prosser and Brown, 1961; Schurmann and Steffensen, 1997).

The prevalence of intraspecific local adaptation (or interdemic variation) may influence the capacity for sub-populations to respond to environmental changes (Grabowski *et al.*, 2009; Tobler *et al.*, 2011; Whitehead *et al.*, 2011). It has previously been proposed that selection pressure for hypoxia tolerance may lead to variation among sub-populations for species with broad habitat ranges (Timmerman and Chapman, 2004). A number of studies to date have found evidence for local thermal adaptation in metabolic traits between sub-populations of species including killifish (*Fundulus heteroclitus*; Dhillon and Schulte, 2011) and Atlantic cod (*Gadus morhua*; Sylvestre *et al.*, 2007; Grabowski *et al.*, 2009). Local adaptation to hypoxia between sub-populations of fish species across broad spatial scales, however, is less well understood, and no study has investigated this for a higher-level predator.

**Figure 1:**
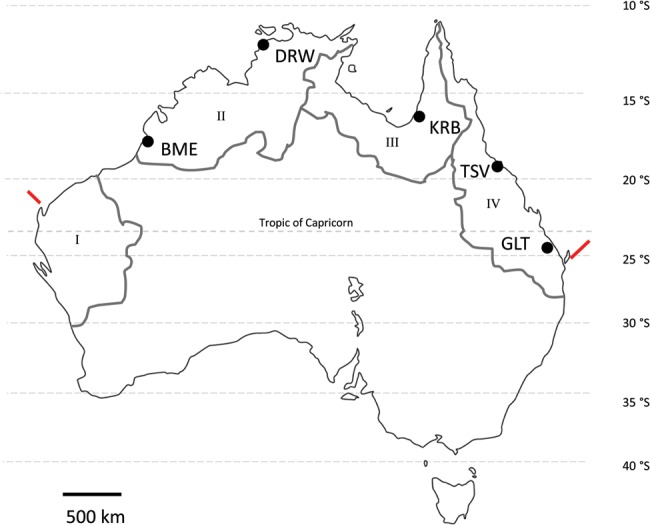
Map of Australia showing where individual sub-populations of barramundi were collected, as follows: BME, Broome; DRW, Darwin; GLT, Gladstone; KRB, Karumba; and TSV, Townsville. Major river drainage divisions of northern Australia have been redrawn from Hutchinson and Dowling (1991) and are indicated as follows: I, Indian Ocean; II, Kimberley and Arafura Sea; III, Gulf of Carpentaria; and IV, North East Coast. Red lines adjacent to the coast indicate the southerly limits of the species' distribution.

To help address this knowledge gap, we investigated [O_2_]_crit_ and resting 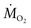 in five different sub-populations of Australian barramundi (*Lates calcarifer*) spanning ∼12° of latitude (Fig. 1). Experiments were performed at two temperatures chosen to represent typical (26°C) and warm summer conditions (36°C). The main aims of this study were as follows: (i) to investigate whether sub-populations differ in their [O_2_]_crit_ and/or resting 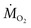; (ii) to quantify how different sub-populations respond to the combined challenges of temperature and hypoxia; and (iii) to determine whether hypoxia tolerance is related to latitudinal position. We expected barramundi sub-populations from lower latitudes to be more hypoxia tolerant than their higher latitude counterparts due to the inverse relationship between water temperature and oxygen solubility. We also expected barramundi (irrespective of sub-population) to exhibit higher metabolic rates at warmer temperatures, and consequently, to be less hypoxia tolerant due to the elevated metabolic demand for oxygen.

## Materials and methods

### Experimental animals and holding conditions

Barramundi juveniles were obtained from five commercial hatcheries located at Broome (Broome Aquaculture Centre), Darwin (Darwin Aquaculture Centre), Karumba (Barramundi Discovery Centre), Townsville (Mainstream Aquaculture), and Gladstone (Gladstone Water Board Barramundi Hatchery; Fig. 1). All five hatcheries use broodstock sourced from local rivers that are separated by a minimum of 700 km (Fig. 1). Wild sub-populations of Australian barramundi differ genetically (Keenan, 1994; Doupé *et al.*, 1999), and broodstock maintained at these locations have been identified as genetically distinct (Smith-Keune C, unpubublished data). Prior to experimental treatments, fish were grown on to a size of ∼200 g over ∼10 months in tanks containing fresh water connected to a recirculating system at the Marine and Aquaculture Research Facility Unit of James Cook University (Townsville, Queensland, Australia). All fish were fed ∼1% body weight per day using a commercial pelleted feed (Ridley Aquafeed, Narangba, Queensland, Australia), and maintained at 26 ± 1°C under a 12 h–12 h photoperiod. Dissolved oxygen was maintained at >75% saturation in the holding tanks, and water quality was monitored daily. Individual fish were weighed and measured prior to experiments and following respirometry. Mean (±SD) body mass and condition factor (*K*) of fish after respirometry were 194.16 ± 22.60 g and 1.17 ± 0.09, respectively. Condition factor was calculated according to the following formula: *K* = 100*a* × *b*^−3^, where *a* = weight (in grams) and *b* = total length (in centimetres; Froese, 2006)_._

### Experimental design

Temperature treatments were selected based on a review of atmospheric and sea-surface temperatures obtained from Australian government databases (AIMS, 2012; BOM, 2012), and from a comprehensive review of river temperatures from previously published data (Pusey *et al.*, 1998; Stuart and Berghuis, 2002; Webster *et al.*, 2005). Based on this information, the two temperature treatments were as follows: (i) a ‘typical’ temperature (26°C), which is representative of the annual mean across the species distribution in Australia and the temperature at which the fish had been held long term; and (ii) a ‘warm’ temperature (36°C), which is regarded to be representative of the upper limit that wild populations experience (Russell and Garrett, 1983), but still within the known tolerance limits for the species (Bermudes *et al.*, 2010). Prior to experiments, fish were removed from their holding tanks and acclimated to one of two temperatures in a separate system where water temperature could be closely controlled. While fish in the 26°C treatment group did not undergo a temperature change relative to prior holding conditions, fish used in the 36°C treatment group were acclimated by increasing the temperature by 1°C per day and then held at 36°C for a minimum of 7 days prior to experimentation. All fish were fed as stated in the previous subsection, but food was withheld for 24 h prior to conducting respirometry to minimize the effect of specific dynamic action on oxygen consumption measurements.

### Respirometry

All measurements were performed using intermittent flow-through respirometry, following best practices outlined in Clark *et al.* (2013). Respirometers (volume = 10.3 l) were fitted with a small Perspex window to allow fish to be observed during respirometry trials. Each respirometer was connected to two pumps; a single recirculating pump to keep water within the chamber mixed, and a flush pump to supply the chamber with aerated water between 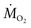 measurements. Respirometers and flush pumps were submerged in a shallow 1000 l tank, with vigorous aeration to provide both stable temperature conditions during experiments (26.2 ± 0.5 and 36.4 ± 0.2°C) and to supply the chambers with ∼100% saturated water during the flush cycle. Temperature-compensated oxygen concentration (in milligrams per litre) of the water within each chamber was continuously recorded (0.5 Hz) using oxygen-sensitive REDFLASH^®^ dye on contactless spots (2 mm) adhered to the inside of each chamber and linked to a Firesting Optical Oxygen Meter (Pyro Science e. K, Aachen, Germany) via fibre-optic cables. Oxygen-sensing equipment was recalibrated daily using a one-point 100% saturation calibration and an electrical (factory-calibrated) zero. Following initial measurements of background respiration in the respirometers, individual fish that had been fasted for 24 h were placed in respirometers in the evening and allowed to acclimate to the respirometers for 16 h. During the acclimation period, the flush pumps attached to each respirometer were set to a 30 min–15 min on–off cycle, and 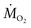 (in milligrams of O_2_ per kilogram per minute) was measured from the decline in oxygen in each respirometer during each 15 min off cycle. Resting 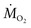 was calculated for each fish as the mean of the lowest three measurements recorded during the acclimation period.

Following the chamber acclimation period and resting 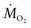 measurements, each flush pump was turned off, and fish were permitted to deplete the oxygen within their respective respirometers down to 5% air saturation. The 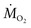 was calculated for each consecutive 5 min period during the decline in oxygen (∼1.5–4 h depending on temperature). Upon reaching 5% air saturation, each flush pump was turned on to restore oxygen levels to 100% saturation. Preliminary trials conducted using similar-sized fish indicated that depletion to 5% saturation was sufficient to calculate [O_2_]_crit_ but still above the oxygen levels that induce loss of equilibrium. The following two values were identified from each [O_2_]_crit_ experiment: (i) pre-hypoxia, 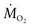, which was calculated from the mean of the lowest three 5 min measurements recorded between 100 and 75% saturation after flush pumps had been turned off; and (ii) [O_2_]_crit_, which was determined using previously established methods (Corkum and Gamperl, 2009; Nilsson *et al.*, 2010). Briefly, [O_2_]_crit_ was determined by fitting two linear regression lines to the measurements (one line based on the calculated pre-hypoxia, 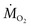 and another line based on the decrease in 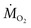 observed during the later stages of the [O_2_]_crit_ test), and calculating the intersection point of the two lines (Ott *et al.*, 1980; Nilsson *et al.*, 2004). After the completion of each trial, additional background respiration measurements were obtained for each chamber in the absence of fish. Any change in background respiration between the start and end of experiments was assumed to be linear when correcting fish 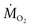.

### Data analyses and statistics

All oxygen consumption rate measurements (in milligrams of O_2_ per kilogram per minute) were calculated using commercial software (LabChart v. 7; ADInstruments, Sydney, NSW, Australia) from the slope of the decline in oxygen concentration according to the formula:

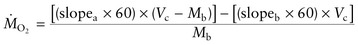

where slope_a_ and slope_b_ are the declines in oxygen (in milligrams per litre per second) measured in the presence and absence of fish within the chamber, respectively, and *V*_c_ and *M*_b_ are the volumes (in litres) of the chamber and fish, respectively.

Resting, 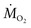 pre-hypoxia, 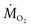 and [O_2_]_crit_ measurements were obtained for 114 barramundi from the five geographically distinct sub-populations, with individual fish considered as an individual replicate for population and temperature treatments. Individual replicates where [O_2_]_crit_ was not calculated (*n* = 7) were excluded from further analyses. The temperature coefficient (*Q*_10_) for resting 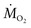 for each population over the 10°C temperature increment was calculated using the formula:

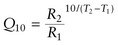

where *R* is resting, 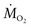 *T* is temperature, 1 represents values at 26°C, and 2 represents values at 36°C.

Statistical analyses were performed using SPSS v. 20 (IBM, Chicago, IL, USA). A general linear model was used to assess the effect of the two main factors (sub-population and temperature) on resting, 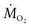 pre-hypoxia 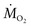 and [O_2_]_crit_. These analyses were followed by one-way ANOVA with Student–Newman–Keuls *post hoc* tests for [O_2_]_crit_ and the non-parametric Kruskal–Wallis test with Dunn's *post hoc* comparison (where the assumption of normality was not met) for resting 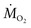 and pre-hypoxia 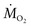 to assess sub-population differences at each temperature (Zar, 2010). Homogeneity of variance and normality were assessed using Levene's test and normal quantile–quantile (Q–Q) plot, respectively. Data are presented as means ± SD, and results were considered statistically significant at *P* < 0.05.

## Results

**Figure 2:**
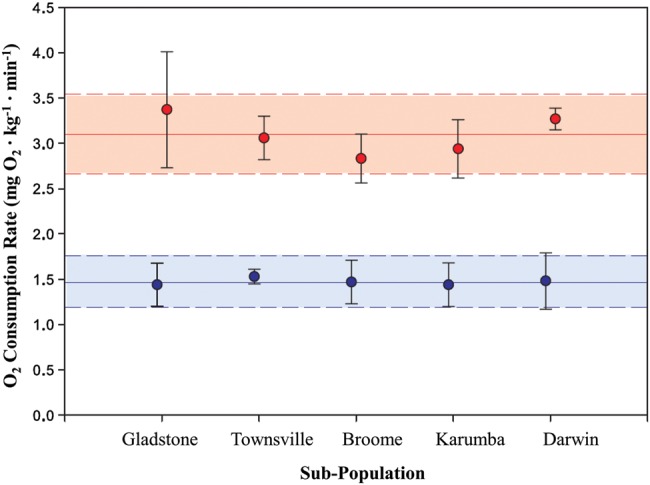
Resting oxygen consumption rates (in milligrams of O_2_ per kilogram per minute; means ± SD) for five barramundi sub-populations at 26°C (blue; *n* = 48) and 36°C (red; *n* = 59). Oxygen consumption rates were calculated from the average slope of the decline in chamber [O_2_] across a 15 min period during the ‘closed’ cycle of intermittent flow-through respirometry, and measured every 45 min. The continous and dashed horizontal lines represent the mean and standard deviation for all populations at each temperature.

Irrespective of population, the resting 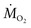 of fish acclimated to 26°C was significantly lower than the resting 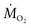 of fish acclimated to 36°C (1.47 ± 0.24 vs. 3.10 ± 0.43 mg O_2_ kg^−1^ min^−1^ for all sub-populations combined; *F*_1, 110_ = 563.57, *P* < 0.001; Fig. 2). There was a significant interaction between sub-population and temperature for resting 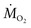 (*P* = 0.025). No differences were found for resting 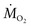 between sub-populations at 26°C (*H*_5_ = 1.446, *P* = 0.84). At 36°C, resting 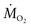 differed from that at 26°C (*H*_5_ = 11.25, *P* = 0.0239; Fig. 2), although Dunn's *post hoc* comparison did not identify differences between individual sub-populations. Pre-hypoxia 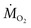 was higher for the Townsville sub-population than for the Darwin sub-population at 26°C (*H*_5_ = 11.90, *P* = 0.018), and was higher for the Gladstone sub-population than for the Broome or Karumba sub-populations at 36°C (*H*_5_ = 17.02, *P* = 0.002). The average *Q*_10_ for resting 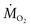 and pre-hypoxia 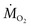 across all populations was 2.12 ± 0.30 and 2.12 ± 0.36, respectively.

**Figure 3:**
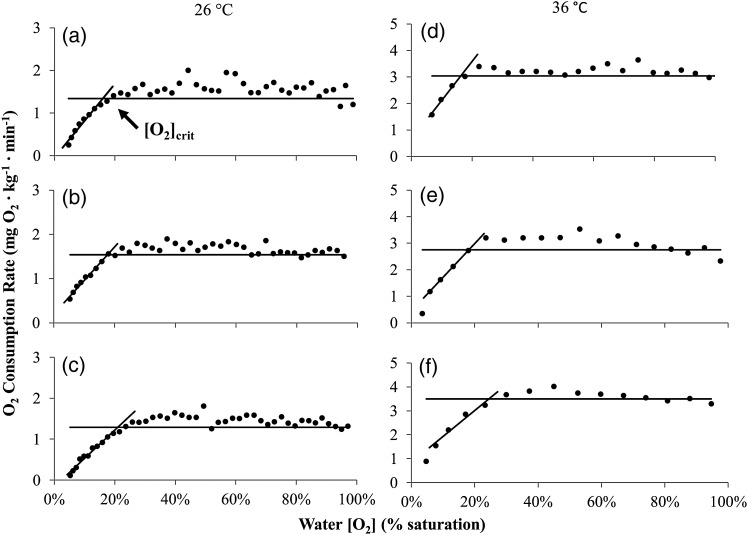
Representative graphs displaying oxygen consumption rate 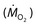 as water oxygen concentration (percentage saturation) decreases for six individuals from three barramundi populations at 26 and 36°C. Populations are indicated by each panel as follows: Gladstone (**a** and **d**), Broome (**b** and **e**) and Darwin (**c** and **f**). The 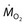 was calculated from the average slope of the decline in chamber [O_2_] for every 5 min period. The critical oxygen saturation ([O_2_]_crit_), indicated by the arrow in the top left panel, was calculated from the intersection between two linear regressions; one for pre-hypoxia 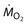, and a second for the steep decline in 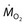 during the later stages of the [O_2_]_crit_ test. Fish took ∼4 h to deplete oxygen within the chambers at 26°C and ∼1.5 h at 36°C. The difference in the vertical scale for fish at 26 and 36°C reflects the increased 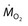 of barramundi at the higher temperature.

**Figure 4:**
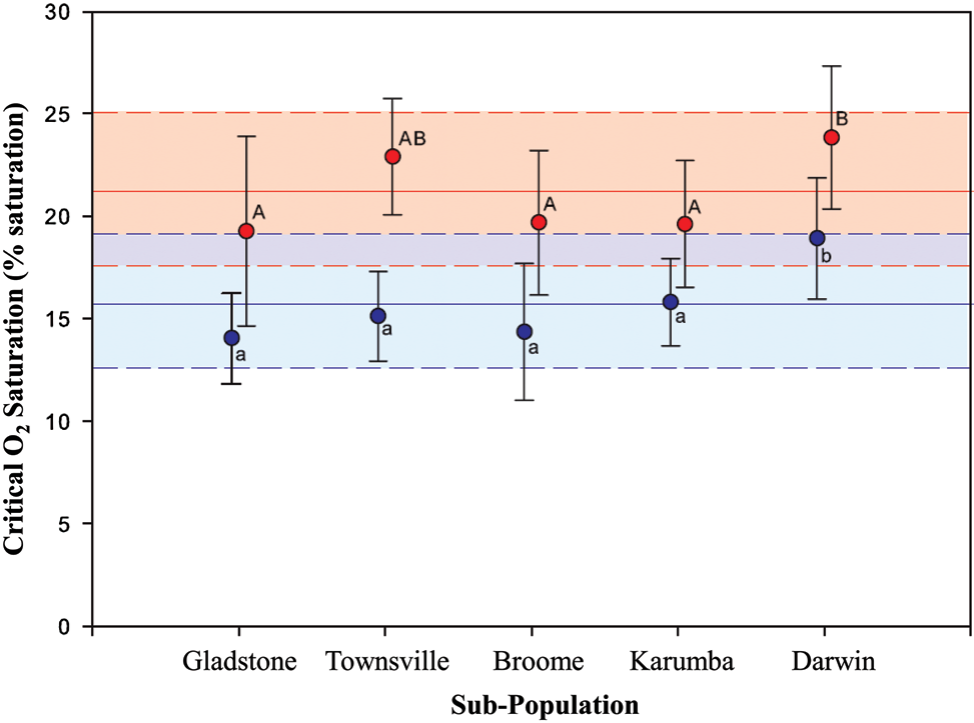
Mean critical oxygen saturation (percentage saturation; mean ± SD) for five sub-populations of barramundi at 26°C (blue; *n* = 50) and 36°C (red; *n* = 63; populations given in Fig. 1). The continuous and dashed horizontal lines represent mean and standard deviation, respectively, of critical oxygen saturation for all populations at each temperature. Letters indicate significant differences (*P* < 0.05; a < b and A < B).

Barramundi exhibited a common and clear trend in response to decreasing oxygen, with fish maintaining a relatively constant 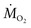 above the [O_2_]_crit_, followed by a steep decline (Fig. 3). There was no significant interaction between population and temperature for [O_2_]_crit_ (*P* = 0.486). Temperature had a significant effect on mean [O_2_]_crit_ across all populations, with lower [O_2_]_crit_ at 26 than at 36°C (*F*_1,114_ = 69.97; *P* < 0.001; Fig. 4). No significant differences were observed for mean [O_2_]_crit_ between four of the five sub-populations tested at both 26 and 36°C (Fig. 4). The exception was that mean [O_2_]_crit_ was highest for fish from Darwin at both 26 and 36°C (18.91 ± 2.97 and 23.84 ± 3.49%, respectively). Critical oxygen saturation measurements varied between individuals, with minimal and maximal values spanning 8.63–23.02% saturation at 26°C (mean = 15.44 ± 3.20% saturation) and 13.47–31.17% saturation at 36°C (mean = 21.07 ± 3.92% saturation). Fish displayed signs of distress, such as erratic movements and pale body colour, below [O_2_]_crit_; however, all fish recovered fully upon returning to 100% saturated conditions.

## Discussion

Species inhabiting thermally stable environments (e.g. equatorial or polar) are thought to be physiologically constrained within a narrow range of environmental thresholds, suggesting that they may be particularly susceptible to increasing temperatures and increasing variability in mean temperatures (Tewksbury *et al.*, 2008). Due to the inverse relationship between temperature and oxygen solubility in water (Diaz and Breitburg, 2009), we expected barramundi from lower latitudes to be more hypoxia tolerant than their higher-latitude counterparts. Contrary to expectations, Darwin fish (low latitude) displayed a slightly higher average [O_2_]_crit_ (less hypoxia tolerant) than fish from the other four populations tested at both control and warm temperatures. However, on the whole, we were unable to find consistent and conclusive evidence for local adaptation in hypoxia tolerance between Australian barramundi sub-populations separated by large longitudinal and latitudinal distances.

Interspecific variation in hypoxia tolerance, as derived from [O_2_]_crit_ measurements, exists for a number of temperate and tropical fish species at temperatures that are routine or typical (Table 1). Species such as Nile tilapia (*Oreochromis niloticus*) can regulate 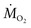 down to 11 ± 3% saturation, whereas other species, such as Atlantic salmon (*Salmo salar*), can regulate 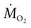 to only 36 ± 3% saturation (Table 1). Schofield and Chapman (2000) reported a [O_2_]_crit_ of 15% saturation for juvenile Nile perch (*Lates niloticus*) at 20°C, and also noted the tendency of Nile perch to congregate at the surface layer of water during periods of aquatic hypoxia in an attempt to enhance oxygen uptake from the more aerated surface waters (commonly referred to as aquatic surface respiration). It has been suggested, however, that Nile perch are inefficient at using this technique to obtain oxygen (Schofield and Chapman, 2000). Anecdotal reports exist of farmed barramundi aggregating at the surface during periods of low DO; however, the capacity for barramundi to use aquatic surface respiration remains unclear. No attempt was made to measure aquatic surface respiration in this study; however, future experiments using respirometers with an air pocket at the surface would help to elucidate the potential for aquatic surface respiration in barramundi, and therefore their capacity to exist for extended periods in hypoxic waters below [O_2_]_crit_.

**Table 1:** Critical oxygen tension ([O_2_]_crit_; expressed as percentage saturation) measurements for a range of temperate and tropical fish species

Species	[O_2_]_crit_ (% saturation)	Temperature (°C)	Fish mass (g)	Authors
*Salmo salar*	36 ± 3	18	151 ± 41	Barnes *et al.* (2011)^a^
	45 ± 4	22		
*Lates niloticus*	15 ± 2	20	4–28	Schofield and Chapman (2000)^b^
*Prochilodus scrofa*	14	25	244 ± 76	Fernandes *et al.* (1995)^b^
	28	35	295 ± 49	
*Anguilla anguilla*	16	25	(yellow phase)	Cruz-Neto and Steffensen (1997)^b^
*Oreochromis niloticus*	11 ± 3	25	301 ± 46	Fernandes and Rantin (1989)^b^
	19 ± 0	35		
*Lates calcarifer*	15 ± 3	26	191 ± 23	Present study
	21 ± 4	36		

^a^Values of [O_2_]_crit_ were converted to percentage saturation from milligrams per litre (100% saturation = 9.4 mg l^−1^ at 18°C and 8.7 mg l^−1^ at 22°C).

^b^Values of [O_2_]_crit_ were converted to percentage saturation from millimetres of mercury (100% saturation = 159 mmHg).

Barramundi are featured in mass-mortality events in northern Australia (Townsend *et al.*, 1992), and previous reports indicate that barramundi succumb rapidly to hypoxia in severe conditions. Pearson *et al.* (2003) reported a ‘lethal DO concentration’ of ∼15% saturation for barramundi living in temperatures ranging from 28 to 30°C; however, the details of the fish and experimental design were unclear, making the results difficult to interpret in the context of the present study. In contrast, Wu (1990) reported no mortality for 150–250 g barramundi held in saltwater after 8 h of exposure to ∼15% saturation at 25°C, but exposure to ∼8% saturation resulted in 50% mortality after 6.5 h. Butler *et al.* (2007) reported an ‘acute asphyxiation concentration’ of 4% saturation for 200 g barramundi in freshwater at 28°C. Fish were visibly distressed in severe acute hypoxia in the present study, but there was only one mortality during the [O_2_]_crit_ tests, despite the fact that each individual fish was exposed to DO of ∼4–5% saturation in order to obtain precise [O_2_]_crit_ measurements.

As well as measuring ‘acute asphyxiation’, Butler *et al.* (2007) investigated the relationship between gill ventilation volume and frequency in response to declining DO for barramundi at 28°C. Butler *et al.* (2007) demonstrated that this species increases ventilation rates down to ∼15–20% saturation, followed by a steep decline in ventilation rate as DO continues to decline. The maximal ventilation rate observed by Butler *et al.* (2007) corresponds closely to our observed [O_2_]_crit_ measurements. This suggests that increasingly elevated ventilatory requirements may be needed to maintain a state of oxygen regulation by the fish in acute hypoxic conditions. Furthermore, a sharp decline in both ventilation rate and 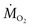 may be inevitable once DO drops below [O_2_]_crit_.

There is varying evidence in the literature for local adaptation of performance traits in sub-populations of Australian barramundi. Cairns (latitude 16°S) and Burrum River (26°S) barramundi have been found to exhibit no differences in specific growth rate, critical thermal minima, or critical thermal maxima between populations (Rodgers and Bloomfield, 1993; Burke, 1994; Keenan, 2000). Recent research, however, has documented differences in performance traits at high temperatures for Australian barramundi from lower (northern) latitudes compared with those from higher (southern) latitudes through the measurement of critical swimming speed (Edmunds *et al.*, 2010) and time to loss of swimming equilibrium when challenged with acute water heating (Newton *et al.*, 2010). Edmunds *et al.* (2012) found elevated transcript abundance of the glycolytic enzyme lactate dehydrogenase-B (*ldh-b*) in southern (Gladstone) sub-populations of Australian barramundi compared with northern (Darwin) sub-populations, and suggested that southern populations may be adapted to cooler temperatures. Differences were observed for pre-hypoxia 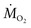 in this study, but such differences were not consistent with resting 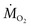, nor was there conclusive evidence for a latitudinal trend. The results from our study indicate that both resting 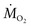 and hypoxia tolerance are conserved across sub-populations of Australian barramundi.

Higher temperatures consistently result in elevated 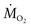 for teleost fish, irrespective of species or life history, reflecting increased metabolic requirements at higher temperatures (Fry and Hart, 1948; Brett and Groves, 1979; Clark *et al.*, 2011). Barramundi elicit a 2-fold increase in resting 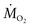 (*Q*_10_ = 2.12) over a 10°C increase in temperature. Higher 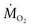 at warmer temperatures might be expected to induce a decrease in hypoxia tolerance in all fish species due to the increased oxygen demands of the fish. Results from previous studies on species such as Nile tilapia (Fernandes and Rantin, 1989; Mamun *et al.*, 2013), rainbow trout (*Oncorhynchus mykiss*; Ott *et al.*, 1980), and common carp (*Cyprinus carpio*; Ott *et al.*, 1980) indicate that [O_2_]_crit_ appears to be less temperature sensitive than what is typically found for resting 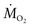; however, this is not consistent across all teleosts. Species such as Atlantic salmon (Barnes *et al.*, 2011), Doederlein's cardinal fish (*Ostorhinchus doederleini*; Nilsson *et al.*, 2010), and Atlantic cod (Schurmann and Steffensen, 1997) elicit a large increase in [O_2_]_crit_ with increasing temperature, particularly towards upper thermal tolerance limits. Our results indicate that [O_2_]_crit_ displays only a small increase for barramundi from typical (26°C) to high temperatures (36°C), demonstrating the resilient nature of this species to the synergistic effects of temperature and environmental hypoxia.

Aside from temperature, a number of other environmental parameters can impact the hypoxia tolerance of fish. The ability to adjust to hypoxic conditions through a lowering of [O_2_]_crit_ following pre-exposure has been reported for the epaulette shark (*Hemiscyllium ocellatum*; Routley *et al.*, 2002) and goldfish (*Carassius auratus*; Fu *et al.*, 2011), although Cook *et al.* (2011) found no differences in [O_2_]_crit_ between naïve and hypoxia-conditioned silver sea bream (*Pagrus auratus*). Henriksson *et al.* (2008) demonstrated that alterations in salinity can increase the [O_2_]_crit_ by up to 30% in prickly sculpin (*Cottus asper*) acclimated to freshwater, compared with fish adapted to seawater; however, the same trend was not observed in the closely related Pacific staghorn sculpin (*Leptocottus armatus*). Barramundi inhabit environments that are prone to acute and chronic hypoxia, and such environments also experience broad fluctuations in salinity and temperature. Currently, there is no understanding of the effects of exposure to repetitive (short-term) or chronic (long-term) hypoxia on the physiology, and consequently, performance of barramundi. However, this topic warrants future research to elucidate more fully the capacity of fishes to tolerate extreme and variable environments.

Elevated temperatures due to climate change are predicted to increase the frequency and severity of hypoxic events through lower O_2_ solubility, increased animal respiration rates, and enhanced stratification (Justic *et al.*, 2001; Diaz and Breitburg, 2009). Such conditions have the potential to increase habitat availability at higher latitudes, while leading to the deterioration of habitats at lower latitudes (Graham and Harrod, 2009; Gardiner *et al.*, 2010). Nutrient inputs to aquatic coastal systems from human waste and agriculture, combined with habitat degradation through increased land use, have the potential to exacerbate further the effects of environmental hypoxia on physiological systems. The present study has shown, for the first time, that a tropical euryhaline fish, barramundi, does not display obvious local adaptation in resting 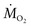 and hypoxia tolerance. This strongly suggests that all sub-populations of this species in northern Australia can cope equally well in environments with large fluctuations in both temperature and dissolved oxygen.
